# Oral Choline Reduced Working Memory-Related Brain Activation in Postmenopausal Women: A Pilot Study

**DOI:** 10.3390/nu18030459

**Published:** 2026-01-30

**Authors:** Julie A. Dumas, Abigail Testo, Anna Senft Miller, Angeles Ozahl, Callum Potts, Jiming Zhang, Marwa Aboukhatwa, James Boyd

**Affiliations:** 1 Department of Psychiatry, University of Vermont, Burlington, VT 05401, USA; abigail.testo@uvm.edu (A.T.); anna.senftmiller@duke.edu (A.S.M.); angeles.ozahl@uvm.edu (A.O.); 2 Colorado Center for Reproductive Medicine, Maimi, FL 33180, USA; callumpotts@gmail.com; 3 Department of Radiology, University of Vermont, Burlington, VT 05401, USA; jiming.zhang@uvmhealth.org; 4 Investigational Pharmacy, University of Vermont Medical Center, Burlington, VT 05401, USA; marwa.aboukhatwa@uvmhealth.org; 5 Department of Neurological Sciences, University of Vermont, Burlington, VT 05405, USA; james.boyd@uvmhealth.org

**Keywords:** choline, menopause, estrogen, working memory, fMRI

## Abstract

**Background/Objectives**: Choline plays an important role in maintaining normal cellular function and overall physiology. Endogenous choline availability depends on the synthesis of phosphatidylcholine via the phosphatidylethanolamine *N*-methyltransferase (PEMT) pathway. Expression of PEMT is influenced by estrogen, as its promoter contains multiple estrogen-responsive elements that enhance enzyme activity. How a low estrogenic condition like menopause influences choline’s effect on the brain is not yet fully understood. **Methods**: In this pilot study, 20 women participated in two study days, with 1650 mg of oral choline bitartrate or a matching placebo administered three hours before a functional and structural magnetic resonance imaging (MRI) scan. Blood oxygen level dependent (BOLD) functional MRI scans were collected on each study day while subjects performed an *N*-back working memory task. **Results**: In this pilot study, no differences in working memory performance were observed, but decreased activation was found for the choline compared to the placebo during the 2-back compared to 0-back conditions in regions of the right temporal lobe (*p* < 0.001 voxel-level threshold, and *p*-FDR < 0.05 cluster-size threshold). When we seeded the right planum temporale to examine its functional connectivity with the rest of the brain, we found that choline modulated a large portion of the working memory network during the difficult memory load condition. **Conclusions**: These results in this pilot study illustrate the effect of choline on working memory-related brain activation and functional connectivity in postmenopausal women. We propose that choline may increase brain functional efficiency in low estrogenic conditions like menopause, but further studies are needed.

## 1. Introduction

Acetylcholine functions as a key signaling molecule in the central and peripheral nervous system and its function has been connected to cognitive changes after menopause as well as to normal and pathological aging [1]. Choline is an essential nutrient that is required for the synthesis of acetylcholine. In addition to its role in the brain, choline has a number of critical structural and physiologic roles throughout the body, including providing structural integrity and signaling function for cell membranes, facilitating lipid transport, and as a source of methyl groups through the diet [2]. When dietary intake is insufficient, choline can be produced endogenously via PEMT-mediated de novo synthesis of phosphatidylcholine [2]. The *PEMT* gene has several estrogen-responsive components in its promoter region and is induced by estrogen [3]. It is notable that estrogen is a major regulator of choline synthesis. What is unknown is the role of choline in influencing brain functioning in the low estrogenic state of menopause.

Cholinergic neurotransmission is reliant on the availability of acetylcholine, which in turn relies on an adequate presence of its substrates: choline and acetyl CoA. Choline is an essential micronutrient found in a variety of membrane-containing food sources, including liver, eggs, and wheat germ [4]. In addition to its importance as a substrate for acetylcholine, choline in the form of phosphatidylcholine is the most abundant phospholipid found in cell membranes, and it may also be irreversibly oxidized to form betaine, a methyl donor for the production of methionine and the eventual methylation of DNA, RNA, and proteins [5]. As such, the availability of choline for use in the cholinergic regulation of cognition is closely interrelated to dietary choline intake, de novo choline production via PEMT, and the use of choline in its other metabolic pathways.

Estrogen appears to be a key regulator of cholinergic neuronal activity in the brain [6,7], and the basal forebrain cholinergic system (BFCS) in particular appears to be dependent on estrogen activity for the maintenance of function over time [8]. Animal studies demonstrated that sudden withdrawal of endogenous estrogen production after ovariectomy was associated with a decline in markers of cholinergic activity, including choline uptake and choline acetyltransferase (ChAT) activity [7], effects that can be reversed by administration of exogenous estrogen [9]. Human studies showed similar beneficial impacts of estrogen therapy for cholinergic functioning. Choline antagonist studies that simulated cholinergic dysfunction showed that estradiol treatment blunted the negative impact of cholinergic blockade, particularly in the domains of attention, vigilance, and psychomotor speed [10]. However, the protective effects of estrogen replacement against a cholinergic antagonist challenge appeared to be limited to younger postmenopausal women [11], supporting the ‘critical period hypothesis’ that early intervention is critical in avoiding irreversible cognitive changes [12].

The current study used a randomized placebo-controlled design to examine whether we could detect a signal in the brain using functional magnetic resonance imaging (fMRI) from a single oral dose of choline bitartrate compared to placebo in healthy postmenopausal women. We hypothesized that we would observe an effect of choline on working memory network activation compared to placebo in healthy postmenopausal women.

## 2. Materials and Methods

All human subject activities described here were approved by the University of Vermont (UVM) Committees on Human Subjects Research Medical Sciences (CHRMS) study number 1720. No human subject activities were conducted before the study approval from UVM CHRMS on 30 October 2020. Each subject gave informed written consent before participating. This mechanistic study of brain functioning began in 2020 and at the time it was not common practice to preregister the study in clinicaltrials.gov. Since that time, we have registered the study, and the study number is NCT07264257.

### 2.1. Participants

Twenty postmenopausal women without cognitive impairment, aged 50–65 years, completed the study. The mean participant age was 58.8 years (SD = 3.6), with additional demographic details provided in Table 1.

Recruitment was conducted via online advertisements targeting the Burlington, VT region. Women were eligible if they were naturally postmenopausal, defined by at least 12 months without menstruation. Menopausal status and MRI eligibility were verified through a telephone screening. Individuals were excluded for factors previously described in related studies, including smoking, prior breast cancer, recent hormone therapy use, or MRI contraindications. No participant reported use of postmenopausal hormone treatment within the past year, and all met telephone screening eligibility requirements.

Eligible participants completed additional screening at the University of Vermont Clinical Research Center (CRC). Following informed consent, participants completed medical and dietary history assessments and underwent physical examinations and laboratory testing to evaluate hematologic, renal, hepatic, and hormonal function. No major medical conditions were identified during clinical evaluation. No subject reported taking choline supplements or following a diet that was high in choline. Subjects were carefully questioned about the history of their perimenopausal and postmenopausal autonomic and vasomotor symptoms, utilizing symptom review and structured menopausal symptom checklists. Participants provided a blood sample that was used to ensure menopausal status of FSH > 20 IU/L. Participants were also cognitively evaluated using the Montreal Cognitive Assessment (MoCA [13]), Brief Cognitive Rating Scale [14], and the Mattis Dementia Rating Scale (DRS [15]) to establish a Global Deterioration Scale score (GDS) which rated the degree of cognitive impairment [14]. Participants were required to have a MoCA score greater than or equal to 26, a DRS score greater than or equal to 123, and a GDS score of 1 or 2.

To assess general neuropsychological functioning similar to our prior work in postmenopausal women, participants completed the Repeatable Battery for the Assessment of Neuropsychological Status (RBANS [16]). The RBANS assessed immediate memory, language, visuospatial/constructional ability, attention, and delayed memory, as well as a global total neuropsychological functioning measure. Working memory was assessed using the letter–number sequencing (LNS) test. Premorbid intellectual functioning was estimated with the test of premorbid function (TOPF), which evaluated reading ability. To confirm cognitively normal status, participants were required to score within one standard deviation of age-adjusted norms on neuropsychological measures, thereby reducing the likelihood of mild cognitive impairment or dementia (see Table 2). A minimum TOPF score of 80 was required for study inclusion.

Behavioral screening included administration of a partial structured clinical interview for DSM-IV-TR (SCID; [17]) to determine the presence or absence of major depressive disorder, mania, or dysthymia. Participants also completed the Beck Depression Inventory (BDI; [18]), with a cutoff score of 10 used to define eligibility. No participants scored over this cut off score during the screening.

**Figure 1:**
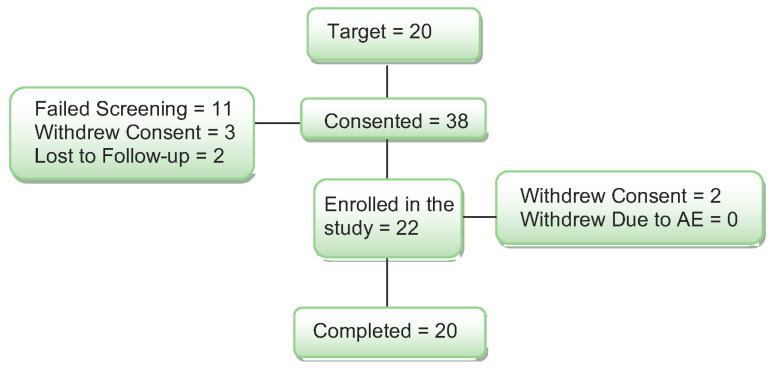
Diagram of subject enrollment and completion through the study.

Thirty-eight women were consented for the study (see Figure 1).

Ten (10) women did not meet the enrollment criteria on the cognitive screening, and they were excluded from the study. One woman was found to be using an estrogen cream that was not reported on the telephone screen, and she was also excluded from the study. Three additional women passed the medical and cognitive screening but were not interested in participating and withdrew consent. Two women were lost to follow-up after screening. Two women started the study and completed one study day. They withdrew from the study for the following reasons: claustrophobia in the MRI and headache after the study day and not tolerating the study procedures. When the blind was broken, both subjects had received the placebo on their study day. Their data were replaced with new subjects for a total of 20 women completing the study.

### 2.2. Challenge Procedure

After passing the medical and psychological screening, subjects came to the UVM CRC for two choline/placebo challenge days. On one day subjects received 1650 mg of oral choline bitartrate in six capsules, and on the second day they received a matching placebo consisting of six capsules of microcrystalline cellulose. A dose of 1650 mg choline bitartrate delivered 1650 mg of choline cation. We chose this dose as it is not high enough to result in side effects but may also result in a signal in the brain functioning measures. Drug administration followed a double-blind procedure, and the sequence of treatments was counterbalanced for all participants. A statistician independent of the study generated the treatment order, which was provided directly to the research pharmacy to maintain blinding of the research team. Each subject took the capsules 180 min (3 h) before the MRI. This time was similar to what has been shown for oral choline to influence brain measures in three prior studies [17,18,19]. The half-life of choline bitartrate is approximately six hours and study days occurred at least 48 h a part. After the MRI session that took approximately 50 min, participants completed further mood and cognitive assessments back at the CRC. After testing, subjects were given lunch. Vital signs and pupil diameter were assessed hourly throughout the day at five time points during the study days. After lunch, subjects were discharged.

### 2.3. fMRI Working Memory Task

During MRI scanning, participants completed a verbal *N*-back task to probe working memory networks. Consonants (excluding L, W, and Y) were presented individually in uppercase on a screen every three seconds. Task difficulty was manipulated across four conditions: 0-back, 1-back, 2-back, and 3-back. In the 0-back condition, participants compared each letter to a predefined target letter. In the higher-load conditions (1-, 2-, and 3-back), they responded whenever the current letter matched the one presented one, two, or three positions earlier in the sequence.

Participants performed each *N*-back level three times in a sequence that was counterbalanced to avoid repeating the same condition twice in a row. Each block consisted of nine trials and lasted 27 s, followed by a 12 s rest marked by a fixation cross (+). The task took 8 min and 12 s to complete. Before drug dosing on each study day, participants completed practice runs to confirm understanding of the task instructions.

Participants responded to each stimulus by pressing buttons on an MRI-compatible fiber optic response system to indicate whether the letter matched or did not match the target. Stimuli were projected using BrainLogics hardware from Psychology Software Tools (Pittsburgh, PA, USA), employing back-projection for cognitive task presentation. Visual stimuli were displayed on an MR-safe monitor. Experimental tasks were programmed with the E-Prime software package version 3.0 (Psychology Software Tools, Pittsburgh, PA USA) and presented via a PC, which also recorded participants’ responses and reaction times. This *N*-back task has been utilized in several prior studies examining brain function in postmenopausal women [20,21,22].

### 2.4. MRI Scan Procedure

All participants underwent scanning on a Philips 3T Achieva d-Stream scanner equipped with a 32-channel head coil. The imaging protocol included T1- and T2-weighted sequences at 0.8 mm isotropic resolution, as well as T2-FLAIR imaging at 1.0 mm isotropic resolution. The T2-FLAIR sequence, designed to detect intracranial pathology, was acquired with a 1.7 mm slice thickness without gaps, a 240 × 240 mm field of view, and a multiband acceleration factor of 3. Structural scans were reviewed by a staff neuroradiologist to identify incidental pathology, and nothing was found in this sample.

Functional MRI parameters included a repetition time (TR) of 800 ms, echo time (TE) of 35 ms, flip angle of 52°, and 2.4 mm isotropic voxel resolution. The field of view measured 216 × 216 × 144 mm^3^, acquired using a multiband acceleration factor of 6 across 60 slices with no interslice gap.

### 2.5. Analysis Methods: Task-Based fMRI

fMRI results presented below were computed using CONN [23] (RRID:SCR_009550) release 22.a [24] and SPM [25] (RRID:SCR_007037) release 12.7771.

MRI Preprocessing: MRI preprocessing of functional and anatomical data was performed using a flexible pipeline [26]. This included realignment with correction for susceptibility distortion interactions, slice timing correction, outlier detection, indirect coregistration to anatomical images, and resampling of functional data onto the cortical surface. Anatomical scans were segmented into gray matter, white matter, and cerebrospinal fluid (CSF) tissue classes using the SPM unified segmentation and normalization algorithm [27,28], employing the default IXI-549 tissue probability map template. Potential outlier volumes were identified with ART [29] based on frame-wise displacement exceeding 0.9 mm or global BOLD signal changes greater than 5 standard deviations [30]. A reference BOLD image was generated for each subject by averaging all non-outlier scans. Functional data were further denoised using a standard pipeline [31], which regressed out confounding signals including five CompCor components from white matter, five from CSF, motion parameters and their first derivatives (12 regressors) [32], outlier scans (fewer than 72 regressors) [30], and linear trends (two regressors) within each run, followed by high-pass temporal filtering of the BOLD time series [33].

First-level GLM analysis: For each participant, Fisher-transformed bivariate correlation coefficients were calculated using a weighted general linear model (weighted-GLM [34]) to estimate the relationship between the BOLD time series of each voxel and the experimental task conditions. Individual scans were weighted using a boxcar function representing each task condition, which was convolved with the SPM canonical hemodynamic response function and rectified.

Group-level analysis: At the group level, a separate general linear model (GLM [35]) was applied to each voxel. Statistical maps were thresholded using a cluster-forming voxel-level threshold of *p* < 0.001 and a cluster-size threshold corrected for multiple comparisons with a family-wise false discovery rate of *p*-FDR < 0.05 [36].

### 2.6. Task-Based Functional Connectivity Analysis

First-level analysis GLM: Seed-based connectivity (SBC) maps were computed to characterize functional connectivity patterns from 132 regions of interest (ROIs) defined by the Harvard–Oxford Brain Atlas. Functional connectivity strength between each seed and target pair was quantified using Fisher-transformed bivariate correlation coefficients derived from weighted-GLM [34], modeling the relationship between their BOLD signal time series. Individual scans were weighted using a boxcar function representing each task or experimental condition, which was convolved with the SPM canonical hemodynamic response function and rectified.

Group analyses were conducted using a GLM [35], with first-level connectivity measures at each voxel serving as dependent variables and group- or subject-level factors as independent variables. Voxel-wise hypotheses were tested using multivariate parametric statistics, incorporating random effects across subjects and sample covariance estimates across multiple measurements. Inferences were made at the level of clusters (contiguous voxel groups). For the seed-to-whole-brain analysis, the right planum temporale from the Harvard–Oxford Brain Atlas in CONN was selected as the seed region. Cluster-level inference was based on parametric statistics derived from Gaussian Random Field theory [37,38], and results were thresholded using a voxel-level cluster-forming threshold of *p* < 0.001 combined with a family-wise FDR-corrected cluster-size threshold of *p*-FDR < 0.05 [36].

### 2.7. Working Memory Performance Analysis

Working memory performance on the *N*-back task was evaluated using signal detection metrics of sensitivity (*d*′) and response bias (C; [39]). Sensitivity (*d*′) quantifies the ability to distinguish between two classes of items, expressed in standard deviation units. In this task, the two classes corresponded to matches and mismatches for each working memory load condition, with higher *d*′ values indicating greater sensitivity and accuracy. Response bias (*C*) reflects a participant’s tendency to classify letters as matches or mismatches, also expressed in standard deviation units. A liberal bias indicates a tendency to classify more items as matches, whereas a conservative bias reflects a tendency to classify more items as mismatches. Bias scores greater than 0 indicate a conservative response pattern, while scores below 0 indicate a liberal response pattern.

### 2.8. Cognitive and Behavioral Measures

After the MRI when subjects returned to the CRC, they completed the Buschke Selective Reminding Task (BSRT) to evaluate episodic memory. In the BSRT a subject was read a list of 16 words and asked to recall all the words. Then on the next trial, they were read the words that they did not recall in the prior trial. Each time the experimenter read the words that were not recalled in the prior trial. This continued for 8 trials or until the subject recalled all 16 words three times in a row. Subjects also completed the Symbol Digit Modalities Test (SDMT [40]) to measure processing speed. Subjects were shown a list of symbols with a corresponding number. They were to fill in the number corresponding to the symbol as quickly and accurately as possible for 90 s. To assess mood and behavior, participants completed the Profile of Mood States (POMS [41]) where each subject judged how well a list of adjectives described their mood at the time of the assessment. The experimenter completed the Brief Psychiatric Rating Scale (BPRS [42]), which was used to measure the presence of psychiatric symptoms after each study day.

## 3. Results

### 3.1. Safety and Tolerability

As described above, two subjects dropped out of the study after reporting headaches on their first study day. One subject was also claustrophobic in the MRI. When the blind was broken, we found that they each received placebo on their study day. No other adverse events were reported by any subject. Thus, one dose of 1650 mg oral choline was safe and well-tolerated by study subjects.

### 3.2. Working Memory Task Activation

First, we examined working memory-related brain activation during the *N*-back task to demonstrate the expected task effect in our sample of postmenopausal women. Second, we examined whether and how choline modulated the working memory network after choline challenge compared to placebo.

**Figure 2:**
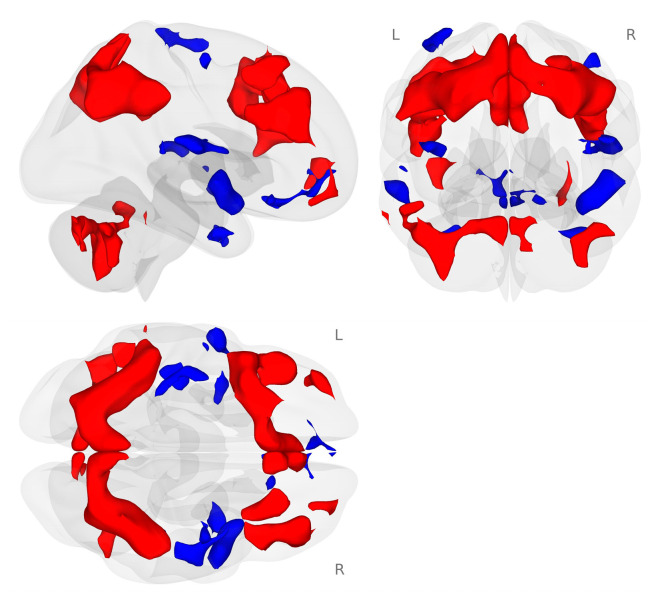
Activation map for the 2-back minus 0-back conditions of the *N*-back tasks (*p* < 0.001 voxel-level threshold, and *p*-FDR < 0.05 cluster-size threshold). The *N*-back tasks activated the expected bilateral frontal, parietal, and cerebellar regions during the placebo challenge day. Red colors represent activation that is greater for the 2-back condition compared to the 0-back condition. Blue colors represent activation that is greater for the 0-back compared to the 2-back condition.

We examined the working memory network generated by the 2-back working memory load condition minus the 0-back match condition during the placebo challenge for all participants. In our sample of healthy postmenopausal women, we found the expected bilateral frontal, parietal, and cerebellar working memory network (Figure 2; [43,44]).

### 3.3. Choline Modulation of Working Memory Activation

**Figure 3:**
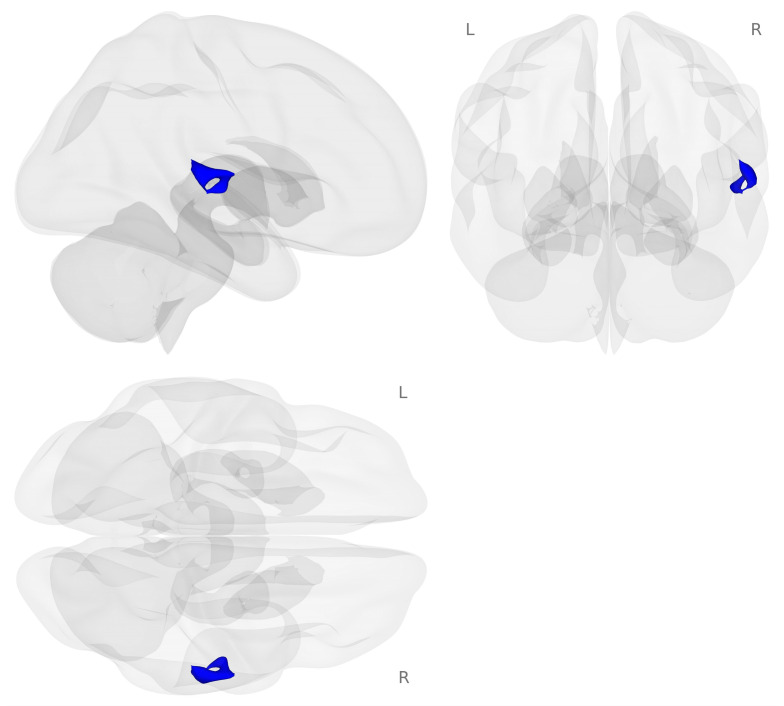
Activation map for the choline minus placebo challenges during the 2-back minus 0-back contrast (*p* < 0.001 voxel-level threshold, and *p*-FDR < 0.05 cluster-size threshold). Blue colors represent decreased activation for the choline challenge compared to placebo during the 2-back working memory load condition.

Next, we examined brain activation for the 2-back minus 0-back contrast on the choline challenge day compared to the placebo challenge day (see Figure 3).

Decreased activation for choline compared to placebo challenge was found in regions of the right temporal gyrus including the right planum temporale, right planum polar, right posterior division of the superior temporal gyrus, and right Heschel’s gyrus (see Table 3).

### 3.4. Task Connectivity

**Figure 4:**
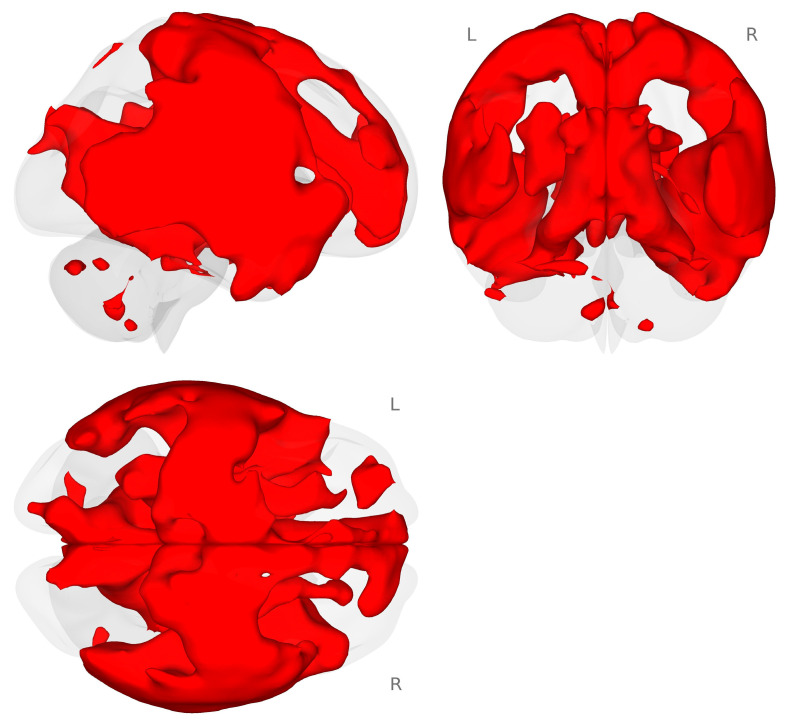
Connectivity map for the right planum temporale during the choline minus placebo challenges for the 2-back minus 0-back contrast (*p* < 0.001 voxel-level threshold, and *p*-FDR < 0.05 cluster-size threshold). Red colors represent increased connectivity for the choline challenge compared to placebo during the 2-back working memory load condition.

Next, we conducted an exploratory analysis of task-based functional connectivity for the planum temporale (PT) to the rest of the brain. We used an atlas-defined seed-to-whole-brain approach to examine the connectivity that occurred during the choline minus placebo and 2-back minus 0-back contrasts. We found that there was a very large and distributed connectivity pattern between the right PT and the rest of the brain (see Figure 4.)

### 3.5. Working Memory Performance

**Figure 5:**
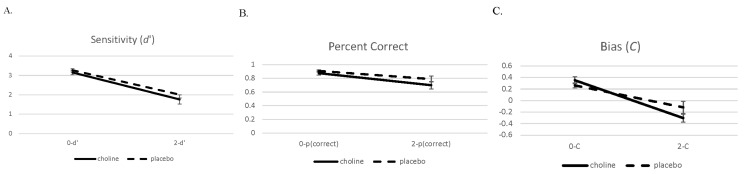
Sensitivity (*d*′ (**A**)), proportion correct (**B**), and bias (*C*, (**C**)) with standard errors on the 0- and 2-back conditions on the choline and placebo challenge days.

We present the performance data on the 2- and 0-back conditions in a similar manner to the brain imaging analysis. Data were analyzed with 2 (challenge: choline and placebo) × 2 (working memory load: 0-back and 2-back) separate mixed model ANOVAs for *d*′, percent correct, and C (see Figure 5). Challenge drug and working memory load were within-subject factors.

The analysis of the accuracy measure *d*′ showed a main effect for working memory load (*F*(1,19) = 165.75, *p* < 0.001). Performance on the 2-back condition was worse than performance on the 0-back condition. There was no main effect or interaction involving challenge drug (*p*s > 0.14). The data pattern for the percent correct measure was similar with the main effect for working memory load (*F*(1,19) = 58.68, *p* < 0.001), with performance in the 2-back condition being worse than the 0-back condition. There was a trend towards an effect of challenge drug (*F*(1,19) = 3.67, *p* = 0.07) and no interaction of challenge drug and working memory load (*F*(1,19) = 1.58, *p* = 0.22).

There was also a main effect for working memory load in the bias measure *C* (*F*(1,19) = 65.24, *p* < 0.001), which showed that participants were more liberal on the 2-back condition compared to the 0-back condition. There was no main effect of challenge drug (*F*(1,19) = 0.76, *p* = 0.40), but there was an interaction involving challenge drug and working memory load for C (*F*(1,19) = 4.78, *p* = 0.04) that indicated that the 2-back condition during the choline challenge resulted in a more liberal performance compared to the other conditions.

### 3.6. Behavioral Measures

After the MRI was completed, participants completed the BSRT, SDMT, and the POMS (Table 4).

Paired *t*-tests showed no choline effects on accuracy measures of the BSRT or the completion and accuracy measures of the SDMT (*p*s > 0.47). The POMS measures also showed no effects of choline on the mood measures (*p*s > 0.16). On the BPRS, no subject on either study day was rated as displaying any psychiatric symptoms.

### 3.7. Vital Signs

Physiological measures—including blood pressure, heart rate, and pupil diameter—were recorded at five time points across the challenge day (Table 5).

Analyses were conducted on the maximum change score from the baseline measurement for each variable. Overall, there were no significant effects of choline challenge on any vital sign measure and the *p* values for systolic and diastolic blood pressure and pulse were >0.40. However, there were trends for an effect of choline on pupil diameter: right pupil: *t*(17) = 1.99, *p* = 0.06; left pupil: *t*(17) = 1.70, *p* = 0.11. The pattern of means showed that pupil diameter decreased more on the choline day than the placebo day (see Table 5).

## 4. Discussion

The current study examined whether functional brain circuitry involved in working memory was affected by a single dose of oral choline in healthy postmenopausal women. The results showed that one dose of choline decreased brain activation primarily in the right temporal regions compared to placebo. Choline had no effect on working memory performance compared to placebo nor did it affect vital signs, mood, or behavior. These findings highlight the importance of menopause status in observing the effects of choline modulation of cognition and that more work is needed to understand choline influences in hypo-estrogenic states.

We found that one dose of choline influenced brain activation patterns during a working memory task in postmenopausal women. Although the effects of choline on working memory performance were not significant, the activation data showed that there was decreased activation for the choline compared to placebo. In our prior studies on estrogen effects on working memory in healthy postmenopausal women, we also found that estrogen treatment resulted in decreased activation compared to a placebo during challenge with a cholinergic antagonist, with no effects on working memory performance [22]. We interpreted that result to demonstrate that estrogen treatment resulted in more efficient brain functioning, as measured by decreased task activation measured with fMRI. Alternative explanations for differences in brain activation between choline and placebo challenge include changes in strategy, habituation to the task, or differences in engagement across conditions or study days. The small sample size makes it difficult to interpret how the brain activation pattern after one dose of choline influences working memory, but the pattern is similar to our prior work with estrogen treatment in postmenopausal women.

The brain regions that showed decreased activation during the choline study day on the more difficult working memory load condition were the planum temporale (PT) as well as the superior temporal gyrus (STG). The PT and STG have been shown to have a role in working memory and they are often involved in auditory working memory tasks (e.g., [45,46]). Buschbaum et al. [45] found that the PT was involved when a task required maintaining both verbal and visual information over a delay, and the STG was activated when the task required rehearsing auditory information in memory. In addition, the PT was functionally connected to the dorsolateral prefrontal cortex, while the STG was functionally connected to the ventrolateral prefrontal cortex. Functional connectivity is a measure of the similarity of activity in different brain regions that is correlated over time [47]. The current study found that the PT and STG were both modulated by choline and were involved in the verbal *N*-back task used here. Interestingly, in an exploratory analysis we found that the atlas-defined PT was functionally connected to a large network including many working memory regions bilaterally. Perhaps this additional widespread connectivity observed in the current study is a result of the addition of the choline challenge to the working memory task. Choline appeared to further engage the working memory network during the more difficult task condition. These functionally connected regions are hypothesized to be working together for information processing or communication across the brain. Thus, choline was involved in modulating these brain regions during the working memory task.

The relationship between choline availability and cognitive functioning has been studied more comprehensively in the context of fetal brain development in animals. Offspring from rodents fed a diet supplemented with choline are found to have up to 30% better performance on visuospatial and auditory memory tasks throughout their lifetime, and demonstrate twice the rate of hippocampal neurogenesis with half the rate of neuronal apoptosis [48,49]. Low-choline diet during pregnancy appeared to reduce methylation potential in the embryonic mouse brain, blocking production of epidermal growth factor receptor (EGFR) protein and disrupting neurogenesis [50]. Data supporting choline supplementation in humans are less conclusive, though recent evidence suggests that choline supplementation at twice the recommended daily intake may be associated with improved infant information processing speed [51]. Other studies have demonstrated that maternal choline supplementation during pregnancy may increase timely development of cerebral inhibition and protect against the later development of attentional problems [52].

Despite growing evidence of its importance at all stages of neurological development, dietary choline intake is inadequate for a significant proportion of the population. A total of 11% of total respondents and just 6% of females in the 2009–2012 National Health and Nutrition Examination Survey achieved adequate choline intake of at least 425 mg/day [53]. PEMT provides an opportunity for de novo production of choline to supplement inadequate dietary intake, though its estrogen-responsiveness limits its ability to overcome severely restricted choline intake in men and postmenopausal women [54]. Even among premenopausal women with an abundance of estrogen availability, common genetic polymorphisms which reduce the estrogen responsiveness of PEMT expose this population to choline deficiency syndrome. Other groups report that approximately 50–75% of women have at least one variant allele in SNPs, leading to decreased PEMT production of choline [50,55].

### Limitations

Several limitations of the present study should be taken into account when interpreting the results. Notably, no significant effect of choline on working memory accuracy was observed. Thus, while we hypothesize that our imaging data may be evidence for choline increasing brain efficiency, further studies of different doses of choline that show performance effects are needed. The small sample size of healthy postmenopausal women in the current study may have affected the ability to detect performance effects of choline on working memory. Additionally, there was only one dose of choline used in the current study and outcome measurements were taken three hours after dosing. A dose–response study is needed to understand how much choline is necessary to influence brain functioning as well as performance. A study with chronic compared to acute dosing is also needed to understand how and whether choline influences working memory in a longer-term manner. In addition, now that we have demonstrated a signal indicating choline has an acute effect on brain functioning, further studies should examine when the maximum effect of choline on brain functioning occurs after dosing. The study design was intended to explore the effect of choline in women with low levels of estradiol (<50 pmol) to minimize the estrogen effect and so it is conceivable that the same effect could be found in men (estrogen < 50 pmol), but this would need to be studied to be confirmed. Future studies should also examine the influence of choline on brain function in those with diseases that impact cholinergic systems like Alzheimer’s disease or Parkinson’s disease.

Additionally, our sample size affected our ability to correct for multiple comparisons. With regard to the brain imaging data, we were not able to correct at an FWE level for the whole-brain analysis. We believe the whole-brain analysis was necessary to examine the influence of choline on working memory-related brain networks in postmenopausal women since these factors have not yet been examined in a placebo-controlled study. We were able to use an FDR correction in this initial study of choline modulation of working memory in postmenopausal women. Thus, we take these data patterns to be suggestive of important relationships between choline, brain activation, and working memory in postmenopausal women and these relationships warrant further study with a larger sample.

Finally, a limitation of this study is that it was not prospectively registered prior to data collection. As a result, hypotheses and analyses were not preregistered. All analyses were specified prior to manuscript preparation, and no analyses were added or removed based on statistical significance.

## 5. Conclusions

Changes in gonadal steroid availability after menopause are associated with physiologic changes that can have profound clinical implications. It has been hypothesized that menopause has detrimental effects on cognition greater than that expected to be seen with normal aging, though evidence for this has been equivocal. The neurobiological processes underlying individual differences in cognition after menopause are not yet fully understood. This study is the first to show modulation of brain functioning in women who are in a low estrogenic state with the nutrient choline that is available in food as well as a supplement over the counter. While further studies are needed to explore whether choline can improve cognition, this initial study provides evidence that one dose of oral choline does indeed affect brain functioning in a potentially beneficial way.

## Figures and Tables

**Table 1:** Demographic data (means and standard deviations) for the postmenopausal women.

	*N* = 20
Age (y)	58.60 (3.6)
Education (y)	16.67 (1.5)
Years since menopause (y)	6.85 (3.6)
Prior estrogen use (N)	5/20
Years of prior estrogen use (y)	0.74 (0.8)
Ethnicity (N)	
Non-Hispanic	20

**Table 2:** Screening and neuropsychological assessment measures (means and standard deviations) for the postmenopausal women, *N* = 20.

	M(SD)	Min	Max
MOCA	28.20 (1.4)	26	30
BDI	3.44 (2.7)	0	7
TOPF	119.85 (7.7)	103	127
LNS	11.80 (2.0)	8	15
RBANS: Immediate Memory	109.00 (8.2)	94	126
RBANS: Visuospatial/Constructional	115.90 (13.1)	89	131
RBANS: Language	112.39 (13.1)	84	134
RBANS: Attention	112.9 (12.8)	85	138
RBANS: Delayed Memory	108.40 (11.5)	81	129
RBANS: Total	117.20 (13.1)	93	142

**Table 3:** Effects of choline compared to placebo for the 2-back minus 0-back contrast including MNI coordinates, cluster size, region descriptions, and voxel-level *p* values.

Contrast	Coordinates x, y, z	Cluster Extent	Region Description	*p* Value
2-back − 0-back				
Choline–Placebo challenge				
	58, −24, 10	83	Right planum temporale	<0.001
	58, −28, 2	15	Right superior temporal gyrus, posterior division	<0.001
	60, −14, 10	11	Right central opercular cortes,	<0.001
	52, −18, 6	7	Right Heschl’s gyrus	<0.001

**Table 4:** Means (standard deviations) for the cognitive and mood assessments post MRI. For two subjects on the choline challenge day, the POMS was not completed because of computer error.

	Choline (M (SD))	Placebo (M (SD))
BSRT: Total Recall	85.16 (15.0)	86.00 (13.2)
BSRT: Recall Consistency	51.11 (19.3)	51.95 (16.6)
BSRT: Recall Failure	10.79 (8.2)	12.15 (7.7)
SDMT: Completed	54.95 (10.0)	55.25 (10.2)
SDMT: Completed Correct	54.20 (10.6)	54.8 (10.3)
POMS	*N* = 18	*N* = 20
Total Mood Disturbance	5.85 (18.9)	2.85 (10.5)
Anger/Hostility	42.39 (5.3)	40.40 (2.2)
Confusion/Bewilderment	7.61 (4.1)	7.00 (2.8)
Depression/Dejection	2.22 (3.6)	1.45 (1.8)
Fatigue/Inertia	5.67 (3.9)	5.00 (2.2)
Tension/Anxiety	6.00 (3.7)	5.65 (3.2)
Vigor/Activity	17.89 (5.4)	17.10 (5.9)
Friendliness	15.72 (2.9)	15.80 (3.1)

**Table 5:** Differences from baseline in vital sign measures for the choline and placebo challenge days.

	Choline	Placebo
Systolic BP (mmHg)	18.80 (11.5)	10.47 (11.2)
Diastolic BP (mmHg)	5.30 (4.1)	4.84 (7.0)
Heart Rate (bpm)	−3.15 (4.0)	−4.32 (4.5)
Pupil, right (mm)	−0.35 (1.0)	0.12 (0.3)
Pupil, left (mm)	−0.35 (1.0)	0.05 (0.4)

## Data Availability

The raw data supporting the conclusions of this article will be made available by the authors on request.

## Abbreviations

The following abbreviations are used in this manuscript:

PEMT	Phosphatidylethanolamine-*N*-methyltransferase
MRI	Magnetic resonance imaging
BOLD	Blood oxygen level dependent
fMRI	Functional magnetic resonance imaging
DNA	Deoxyribonucleic acid
RNA	Ribonucleic acid
BFCS	Basal forebrain cholinergic system
ChAT	Choline acetyltransferase
CHRMS	Committees on Human Subjects Research Medical Sciences
M	Mean
SD	Standard deviation
UVM	University of Vermont
CRC	Clinical Research Center
FSH	Follicle stimulating hormone
MoCA	Montreal cognitive assessment
DRS	Dementia rating scale
GDS	Global deterioration scale
RBANS	Repeatable Battery for the Assessment of Neuropsychological Status
LNS	Letter–number sequencing
TOPF	Test of premorbid functioning
SCID	Structured clinical interview for Diagnostic and Statistical Manual four revised
BDI	Beck Depression Inventory
MR	Magnetic resonance
PC	Personal computer
T1	Transverse relaxation time
T2	Longitudinal relaxation time
FLAIR	Fluid inversion recovery
CONN	Functional connectivity toolbox
SPM	Statistical parametric mapping
ART	Movement-related Artefacts
CSF	Cerebrospinal fluid
GLM	General linear model
FDR	False discovery rate
SBC	Seed-based connectivity
ROI	Region of interest
RRC	ROI to ROI connectivity
*d*′	Sensitivity
*C*	Bias
BSRT	Buschke selective reminding task
POMS	Profile of mood states
BPRS	Brief psychiatric raring scale
PT	Planum temporale
ANOVA	Analysis of variance
STG	Superior temporal gyrus
EGFR	Epidermal growth factor receptor
SNP	Single-nucleotide polymorphism
